# Enhancing Interfacial Dioxygen Bridging Dynamics of Waste‐Derived Cathode Catalysts for Augmented High‐Rate Performance in Li‐O_2_ Batteries

**DOI:** 10.1002/advs.202522315

**Published:** 2026-01-12

**Authors:** Jixiong Zhang, Shuxuan Ma, Hengfeng Liu, Binbin Huo, Yunbo Wang, Zhihui Sun, Kai Zeng

**Affiliations:** ^1^ State Key Laboratory of Intelligent Construction and Healthy Operation and Maintenance of Deep Underground Engineering China University of Mining and Technology Xuzhou China; ^2^ School of Mines, Key Laboratory of Deep Coal Resource Mining of Ministry of Education University of Mining and Technology Xuzhou China; ^3^ Institute of Smart City and Intelligent Transportation Southwest Jiaotong University Chengdu China

**Keywords:** electronic structure, fine slag, interfacial dioxygen bridge, Li‐O_2_ battery

## Abstract

Lithium–oxygen batteries (LOBs) are widely regarded as promising candidates for next‐generation high‐specific‐energy storage systems. However, their development is hindered by the disorderly accumulation of insulating products and sluggish oxygen redox kinetics (including ORR and OER). The rational design of high‐efficiency cathode catalysts is therefore critical for improving the overall performance of LOBs. In this work, a novel concept of interfacial dioxygen bridge coupling to design bifunctional cathode electrocatalysts derived from mining solid waste is introduced. Specifically, lattice distortion induced by oxygen vacancies, together with the intrinsic charge repulsion of Ti–O–C bonds on the (1–20) plane, cooperatively drives surface reconstruction and refines the local coordination environment. This structural adjustment consequently enhances the adsorption properties for ORR/OER, thereby improving the overall electrochemical performance. The Fine Slag/Ti_4_O_7_@TiC hybrid exhibits modified chemical bonding and remarkable stability at high rates. LOBs fabricated with the Fine Slag/Ti_4_O_7_@TiC catalyst deliver a reduced voltage polarization of only 1.42 V, ultralong cycle stability of 210 cycles at 3000 mA g^−1^, and outstanding high‐rate performance (from 1000 to 10 000 mA g^−1^). DFT analysis and ex‐situ characterization further elucidate the pivotal role of interfacial dioxygen bridge coupling in governing the micro‐chemical composition and manipulating the formation pathway of Li_2_O_2_.

## Graphical abstract

This study fabricates Fine Slag/Ti_4_O_7_@TiC hybrids with interfacial dioxygen bridge coupling to optimize oxygen redox dynamics in lithium‐oxygen batteries (LOBs). Lattice distortion from oxygen vacancies and inherent charge repulsion of Ti‐O‐C interfacial coupling reconstruct and refine the surface coordination environment. LOBs using this catalyst exhibit ultralong cycle stability (210 cycles at 3000 mA g^−1^) and ultra‐high rate performance (1000–10 000 mA g^−1^).

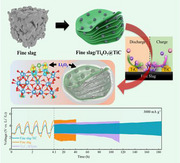

## 1 Introduction

The rapid progression of energy storage technology is crucial for the seamless integration of renewable energy sources. However, the capacity‐limited lithium‐ion batteries struggle to meet diverse needs [1]. Li‐O_2_ batteries (LOBs) have emerged as promising alternatives by virtue of their remarkable high energy density and environmental benefits [2]. Conventional nonaqueous LOBs are composed of a lithium metal anode, an aprotic electrolyte, and an oxygen cathode [3]. The battery operates on the electrochemical reaction 2Li + O_2_ ↔ Li_2_O_2_, with a voltage of 2.96 V relative to the Li/Li^+^ reference. During discharge, molecular oxygen is reduced at the cathode to form lithium peroxide (Li_2_O_2_). Conversely, during charging, Li_2_O_2_ is oxidized, reverting back to oxygen and lithium ions [4]. This semi‐open configuration of LOBs yields an impressive theoretical energy density of approximately 3600 Wh kg^−1^. Nevertheless, the commercial viability of LOBs is substantially impeded by several limitations, including reduced actual discharge capacity, inferior rate capability, and low cycle stability [5, 6]. These drawbacks could be mainly related to the slow oxygen reduction kinetics, the vigorous nucleophilic reactivity of superoxide intermediates, and the excessive deposition of insulating and non‐soluble discharge byproducts, which all occurs at the cathode surface [7, 8]. Therefore, the precise design of cathode materials is essential to address the existing obstacles in lithium‐oxygen battery technology [9, 10].

Characterized by ultra‐thin atomic layer structure and large aspect ratio, 2D layered materials exhibit exceptional performance in electrocatalysis [11]. MXenes deliver various desirable properties, including high density, excellent electrical conductivity, robust mechanical strength, and tunable surface functional groups [12]. These attributes position MXenes as a promising material for electrochemical energy storage applications. MXenes could establish a stable and conductive biphasic interface with the discharge product Li_2_O_2_, demonstrating superior catalytic performance in LOBs [13]. Besides, The highly active functional groups on the surface of MXenes generate additional reaction sites, thereby enhancing the specific capacity of LOBs [14]. Additionally, MXenes have a controllably large interlayer spacing that facilitates rapid and reversible energy storage processes [15]. However, metastable Ti atoms on the MXene surface may occupy active sites and alter local electronic states, which is unfavorable for the adsorption and desorption of reaction intermediates in LOBs [16]. MXene might trigger structural reconstruction and aggregation, thereby diminishing the electrocatalytic activity [17]. Transition metal carbide TiC is commonly referred to as interstitial alloys, which are compounds formed by the insertion of carbon atoms into the interstitial sites of Ti. The insertion of carbon atoms causes an increase in the atomic spacing between the metal atoms, which in turn leads to the broadening of the Ti d‐band. The broadened d‐band raises the electron state density near the Fermi level, thereby conferring upon transition metal carbides a catalytic activity akin to that of noble metals. Moreover, Porous TiC cathode catalyst could markedly reduce the occurrence of side reactions caused by electrolyte decomposition in comparison to carbon‐based materials in LOBs [18].

The utilization of coal gasification fine residues (CGFRs) represents a critical practical challenge in the domains of environmental conservation and resource recycling. Coal gasification fine residues are generated as solid waste during the coal gasification process, which consist of unreacted carbon and ash fractions including inorganic minerals inherent [19]. Currently, the utilization of coal gasification fine slag is primarily directed toward the fabrication of functional materials from the slag's high‐content residual carbon. The synthesis of porous materials from coal‐based solid waste could achieve cost reduction and promote waste‐to‐value conversion. Li et al. discovered that the carbon content in gasification fine slag is elevated at 39%, which delivers graphitized structure [20]. The presence of graphitic carbon could enhance the dielectric properties of carbon materials, endowing them with the capability for microwave absorption. Guo et al. synthesized porous carbon from the residual carbon in coal gasification slag by a combination strategy of acid etching and ammonia activation. The porous carbon demonstrated exceptional performance in ORR with a low potential of 0.68 V [21]. Moreover, the porous carbon was effectively utilized as the air cathode in the zinc‐air battery, achieving a power density of 88 mW cm^−2^ at a current density of 154.9 mA cm^−2^. However, there are still many deficiencies in the utilization of residual carbon in coal gasification fine slag at present. On the one hand, the intricate composition and structure of the residual carbon present considerable difficulties in extraction and modification. On the other hand, the maturity of existing conversion technologies is insufficient for the efficient conversion of this carbon into energy‐storage‐compatible forms. Consequently, the urgent development of novel strategies and advanced methods to augment the utilization of residual carbon in these residues, especially for energy storage, is essential to foster sustainable industrial growth.

In this work, we first introduce a novel concept of interfacial dioxygen bridge coupling to design bifunctional cathode electrocatalysts derived from mining solid waste. Specifically, the lattice distortion induced by oxygen vacancies and the charge repulsion inherent in the Ti‐O‐C bonds in (1–20) plane result in the reconstruction and refinement of the surface local coordination environment. This structural adjustment consequently enhances the adsorption properties for ORR/OER, thereby improving the overall electrochemical performance. Fine Slag/Ti_4_O_7_@TiC hybrid exhibits modified chemical bonding and remarkable stability at high rates. LOBs fabricated with Fine Slag/Ti_4_O_7_@TiC catalyst deliver reduced voltage polarization of only 1.42 V and ultralong cycle stability of 210 cycles at 3000 mA g^−1^, and ultra‐high range rate performance (from 1000 to 10 000 mA g^−1^). DFT analysis and ex‐situ characterization techniques elucidate the pivotal role of interfacial dioxygen bridge coupling in governing the micro‐chemical composition and manipulating the formation pathway of Li_2_O_2_.

## 2 Results and Discussion

### 2.1 Morphological and Structural Characterization

**FIGURE 1:**
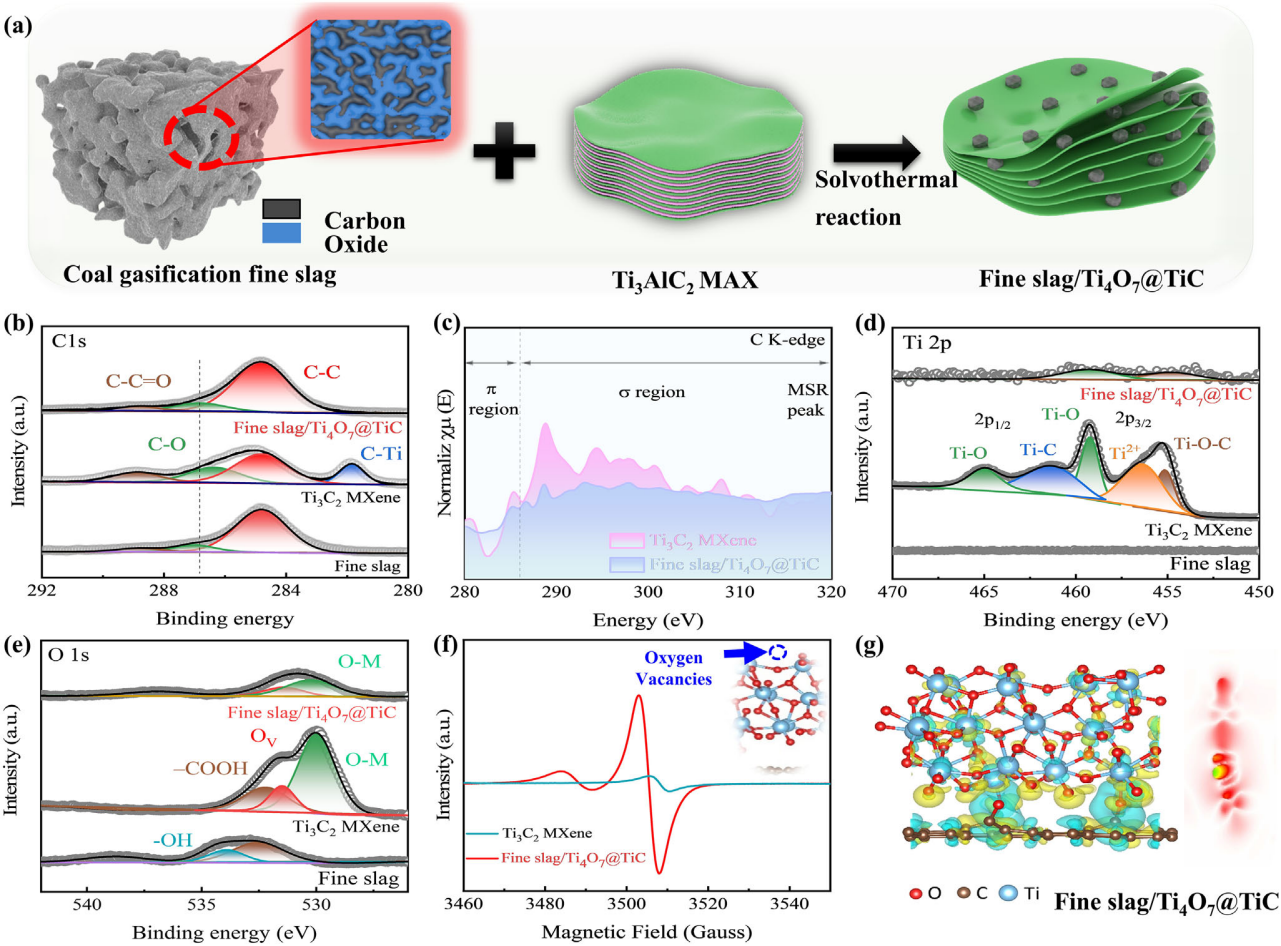
(a) Schematic illustration of the synthesis process for Fine Slag/Ti_4_O_7_@TiC; (b); High‐resolution C 1s of various catalysts; (c) Carbon K‐edge spectra of Fine Slag/Ti_4_O_7_@TiC and Ti_3_C_2_ MXene; High‐resolution Ti 2p (d), and O 1s (e) XPS spectra of various catalysts; (f) EPR spectra of the synthesized catalysts; (g) Charge density difference plots for Fine Slag/Ti_4_O_7_@TiC, where yellow indicates regions of increased charge and cyan denotes areas of decreased charge.

As depicted in Figure 1a, the fine slag derived from coal gasification is predominantly composed of carbon and multiple metal oxides, boasting a fixed carbon content exceeding 25% (Figure S2). The fine slag is modified by controlled acid leaching process to eliminate the multi‐metal oxides, thereby maintaining the integrity of the residual carbon. This approach could guarantee the separation of porous carbon from the slag matrix without compromising its structural integrity. Subsequently, with the high‐pressure hydrothermal reaction and strong high‐fluorine coordination, fine slag could interact with Ti_3_AlC_2_ Max to facilitate the synthesis of the desired composite (Fine Slag/Ti_4_O_7_@TiC). The XRD spectrum of fine slag in Figure S3 delivers strong diffraction peaks located at 26.9° and 21.1°, which could be identified as quartz. Additionally, the fine slag exhibits a “hump‐like” peak between 20° and 40°, suggesting the presence of amorphous aluminosilicate phases. These amorphous glassy phases play the role of effective carriers for trace elements and carbonaceous. Furthermore, the Ti_3_C_2_ MXene exhibits broad diffraction peaks within the 10–80°, revealing its amorphous characteristics. In contrast, Fine Slag/Ti_4_O_7_@TiC shows distinct characteristic diffraction peaks attributed to Ti_4_O_7_ (JCPDF: #50–0787) and TiC (JCPDF: #32–1383). In addition, there is no detection of carbon materials and multi‐metal oxides, indicating that the multi‐metal oxides in the fine slag have been effectively removed, and the carbon materials maybe maintained within an amorphous structure. XPS testament was performed to precisely analyze the surface elemental composition, chemical states, and electronic binding energies of Fine Slag/Ti_4_O_7_@TiC. The high‐resolution spectrum of C 1s in Figure 1b illustrates that all catalysts deliver dominant three peaks corresponding to C‐C, C‐O, and C‐C = O. Obviously, Ti_3_C_2_ MXene exhibits a characteristic peak corresponding to C‐Ti, indicating the successful synthesis of the MXene's distinctive chemical composition in the relevant experiment. Besides, the increase of C‐C bonding in the skeleton of Fine Slag/Ti_4_O_7_@TiC leads to a higher density of C atoms at surface sites, leading to enhanced surface activity or reactivity. To further explore the interfacial dioxygen bridging mechanism of Fine Slag/Ti_4_O_7_@TiC, synchrotron radiation analysis was conducted. According to the C K‐edge XAFS spectrum in Figure 1c, a sharp resonance at approximately 285 eV in Fine Slag/Ti_4_O_7_@TiC is attributed to the 1s to π* transition, while a broad band above 290 eV is primarily due to 1s to σ* transitions from carbon atoms with sp2 and sp3 hybridization. Some of the intensity observed between these principal features is associated with the presence of C‐O and C = O bonds. These spectral characteristics are attributed to the existence of oxygen defects and Ti‐O‐C Units within Fine Slag/Ti_4_O_7_@TiC. The high‐resolution Ti 2p spectra of typical Ti_3_C_2_ MXene in Figure 1d could be resolved into five distinct peaks at 465.0, 461.2, 459.2, 456.4, and 455.2 eV, corresponding to Ti‐O (Ti 2p_1/2_), Ti‐C (Ti 2p_1/2_), Ti‐O (Ti 2p_3/2_), Ti^2+^ (Ti 2p_3/2_), and Ti‐O‐C (Ti 2p_3/2_) respectively. Notably, Fine Slag/Ti_4_O_7_@TiC exhibits only two characteristic peaks related to Ti‐O (Ti 2p_3/2_) and Ti‐O‐C (Ti 2p_3/2_), which are attributed to the in‐situ formation of the carbon/Ti_4_O_7_ protective layer. Moreover, a slight downshift (0.6 eV) in Fine Slag/Ti_4_O_7_@TiC could be observed, which is attributed to the strong interaction between carbon and oxygen. This suggests that the distribution of Ti‐related oxygen‐based bonding configurations may be more intricate and less ordered. The high‐resolution O 1s spectrum of Ti_3_C_2_ MXene in Figure 1e could be deconvoluted into three peaks with binding energies of 532.2, 531.4, and 529.9 eV, associated with the ‐COOH, oxygen vacancy (abbreviated as Ov), and O–Metal, respectively. The O 1s spectra for fine slag with acid treated is divided into two distinct peaks of ‐OH and ‐COOH. The disappeared O‐M bonding in fine slag could further confirm that the metal oxides in fine slag have been completely removed. However, there is no functional group bonding in Fine Slag/Ti_4_O_7_@TiC. Compared with Ti_3_C_2_ MXene, the relative content of O_v_ increases in Fine Slag/Ti_4_O_7_@TiC, indicating that Fine Slag/Ti_4_O_7_@TiC has better electrocatalytic activity including altered electronic structure and improved adsorption ability. To in‐depth analyze the characteristics of oxygen vacancies in as‐prepared samples, electron paramagnetic resonance (EPR) measurement is produced in Figure 1f. Both Fine Slag/Ti_4_O_7_@TiC and Ti_3_C_2_ MXene exhibit a characteristic signal of g = 2.004, indicating the presence of oxygen vacancies. Additionally, The defect signal of Fine Slag/Ti_4_O_7_@TiC is significantly stronger, in agreement with the XPS results. The DFT analysis reveals that C preferentially bonds with O atoms from Ti_4_O_7_ active layer of the Fine Slab/Ti_4_O_7_@TiC hybrid (Figure 1g), resulting in a diminished charge adsorption capacity oxygen (O). Specifically, when C atom is combined with O atoms from Ti_4_O_7_ layer on TiC, the charged areas between the C site and O atoms are observed to be smaller than those between the Ti site and O atoms (highlighted in cyan). The interface oxygen bridge between Ti and C facilitates the formation of chemical bonds and the transfer of electrons/ions between Ti_4_O_7_@TiC and amorphous carbon, thereby improving conductivity and chemical reactivity. The DFT results is consistent with previous XPS analysis, indicates that the Ti‐O‐C coupling is fabricated by the favorable bonding between foreign C atom and Ti_4_O_7_ active layer.

**FIGURE 2:**
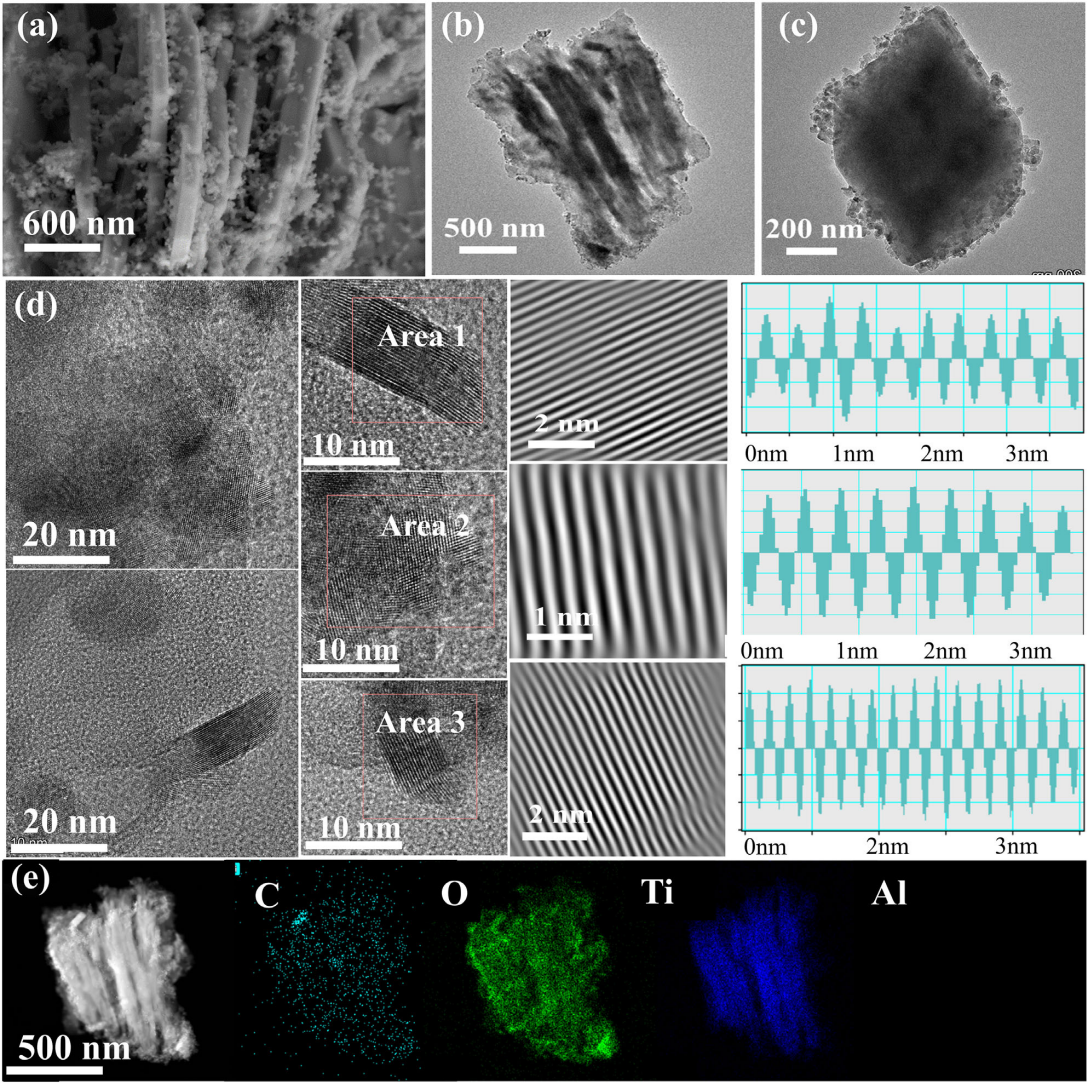
(a) SEM image; (b) TEM image and (c); (d) HR‐TEM image; (e) EDS mapping images of Fine Slab/Ti_4_O_7_@TiC.

The morphologie and microstructure of Fine Slag/Ti_4_O_7_@TiC was examined by scanning electron microscope (SEM) and transmission electron microscope (TEM). As depicted in SEM (Figure 2a; Figures S4 and S5), Fine Slag/Ti_4_O_7_@TiC manifests a distinct nano‐layered structure uniformly dispersed nanoparticles. The unique structure could enhance matrix electrochemical performance by optimizing ion diffusion pathways, and the uniform distribution of nanoparticles provides abundant active sites in facilitating efficient catalytic reactions. Low‐resolution TEM image in Figure 2b,c shows that reveals that the surface of the Fine Slag/Ti_4_O_7_@TiC is not only densely coated with nanoparticles but also encapsulated by a thin shell. The thin shell could potentially play a crucial role in protecting the nanoparticles from external environmental interference and maintaining structure stability. The high‐resolution TEM image in Figure 2d further shows a distinct multiphase coexistence including amorphous and crystalline in Fine Slag/Ti_4_O_7_@TiC. Moreover, the entire crystalline region was identified by characteristic lattice spacing of 0.337 nm, corresponding to the (1–20) crystallographic planes of the Ti_4_O_7_. This analysis confirms that the active protective layer formed in situ on the surface is Ti_4_O_7_. Ti_4_O_7_ demonstrates superior catalytic activity, which could be ascribed to its crystal structure with oxygen defects, and the coordination environment that optimizes the electron cloud density and charge distribution. Besides, amorphous structure could provide abundant unsaturated coordination atoms, thereby offering a substantial number of active sites and exhibiting favorable adsorption for reactants. The atomic‐resolution HAADF images in Figure 2e reveal that Fine Slag/Ti_4_O_7_@TiC is composed of a uniformly distributed aggregate of Ti, C, and O element, further verifying the effective formation of the heterostructure. Notably, carbon is wildly distributed throughout the whole structure, implying the formation of C shell. The absence of lattice fringes corresponding to carbon in the previous high‐resolution TEM images reveal indirectly that the surface carbon coating is amorphous, Figure S11 is the diffraction pattern of Fine Slag/Ti_4_O_7_@TiC. The pattern shows several wide and dispersive diffraction rings, which can directly indicate that the carbon layer is amorphous.

### 2.2 2032‐Coin Li‐O_2_ Battery Performance

**FIGURE 3:**
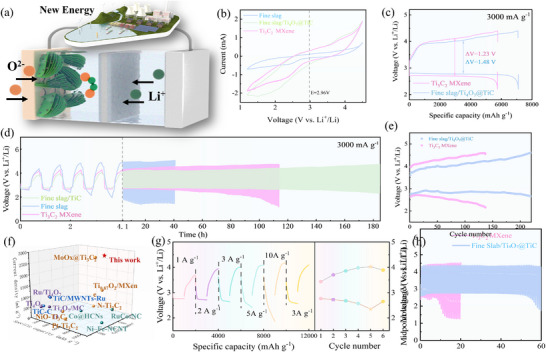
(a) the operational mechanism of LOBs equipped with a Fine Slab/Ti_4_O_7_@TiC‐based cathode; (b) the cyclic voltammetry (CV) curves obtained at a scan rate of 0.1 mV s^−1^ across a voltage range of 1.2–4.4 V; (c) the initial full discharge/charge profiles for the LOBs based on the synthesized catalysts at a current density of 3000 mA g^−1^; (d) the discharge/charge cycling stability of the LOBs utilizing the synthesized catalysts at a current density of 3000 mA g^−1^, with a cut‐off capacity of 1000 mAh g^−1^; (e) the overpotential values for Fine Slab/Ti_4_O_7_@TiC and Ti_3_C_2_ MXene throughout the cycling process; (f) comparative analysis of the performance of LOBs using Fine Slab/Ti_4_O_7_@TiC and other documented electrocatalysts; (g) the rate capabilities of the catalyst‐based LOBs at varying current densities; (h) the discharge/charge cycling stability of the LOBs with the synthesized catalysts at a current density of 3000 mA g^−1^ and a cut‐off capacity of 1000 mAh g^−1^ following the magnification test.

The electrocatalytic efficacy of the fine slag‐based catalyst was rigorously evaluated using 2032‐coin type lithium‐oxygen batteries (LOBs). 2032‐coin type LOBs with well‐defined geometry could provide a reliable platform for assessing the catalyst's activity in facilitating electrochemical reactions in ORR/OER. Through the precise monitoring of parameters including charge and discharge capacities, cycle life, and overpotential, a comprehensive insight into the electrocatalytic activity and stability of the fine slag‐based catalyst was gained. Figure 3a demonstrates graphically the complete working mechanism of LOBs. During the discharge process, external oxygen interacts with lithium ions (diffusing from the Li‐anode), leading to the formation of lithium‐based products on the cathode; Reversibly, these products are subsequently decomposed following recharging. The catalyst‐fabricated cathode plays a dual role as an active site for electrochemical reactions and for the storage/decomposition of reaction products. Therefore, the catalytic activity of cathode catalyst is pivotal in enhancing the practical efficiency of LOBs. As depicted in the cyclic voltammetry (CV) curves (Figure 3b), the Fine Slab/Ti_4_O_7_@TiC hybrid exhibits a higher ORR onset potential and a lower OER onset potential, indicative of superior ORR/OER bifunctional catalytic activity. Specifically, the Fine Slab/Ti_4_O_7_@TiC hybrid delivers a larger anodic peak area, which suggests a higher specific capacity. The full charge‐discharge curve of LOBs could yield key information including voltage variation trends, charge‐discharge capacities, and polarization degrees. Compared with monomer Ti_3_C_2_ Mxene, the Fine Slab/Ti_4_O_7_@TiC catalyst material exhibits superior full‐charge‐discharge specific capacity(7093 mAh g^−1^) and lower voltage polarization(1.23 V) in Figure 3c. To investigate the potential applications of electrocatalysts derived from mining solid waste, the cycling stability of LOBs was assessed at a current density of 3000 mA g^−1^ within a constrained specific capacity of 1000 mAh g^−1^. As depicted in Figure 3d, the unadulterated fine slag catalyst exhibits the biggest voltage polarization and the poorest endurance stability in LOBs, indicating a lack of substantial electrocatalytic activity. By comparison, the LOBs incorporating the Fine Slab/Ti_4_O_7_@TiC catalyst demonstrate stable performance over 210 cycles (equivalent to 180 h) without significant degradation at an ultrahigh current density of 3000 mA g^−1^. Moreover, the Fine Slab/Ti_4_O_7_@TiC catalyst maintains a consistently low and stable overpotential of 1.42 V during the long‐term cycling of LOBs in Figure 3e, in contrast to Ti_3_C_2_ MXene which shows significant voltage polarization (∼2.12 V). Rate testing is conducted to evaluate the dual functional stability of solid waste‐based catalyst in structure and activity. To our knowledge, the comprehensive endurance stability of Fine Slab/Ti_4_O_7_@TiC at different current densities and cut‐off capacities was surpassed that of most recently reported related cathode catalysts in LOBs in Figure 3f. It could be attributed to the robust interaction at carbon/Ti_4_O_7_/TiC ternary heterointerface, which leads to enhanced coordination optimization and electronic compensation, thereby facilitating electron/ion transport and reducing overpotentials [14, 15, 16, 17, 21, 22, 23, 24, 25, 26, 27, 28, 29]. Figure 3g illustrates that the Fine Slab/Ti_4_O_7_@TiC catalyst exhibits minimal variation in performance across a current densities range from 1 to 10 A g^−1^. Specifically, the catalyst sustains a consistent discharge‐charge overpotential, varying from approximately 0.67 V at 1000 mA g^−1^ to around 1.68 V at 10 000 mA g^−1^, and returning to about 1.24 V when the current density is reduced back to 2500 mA g^−1^. In contrast, the LOB equipped with a Ti_3_C_2_ MXene catalyst exhibits more pronounced polarization under ultrahigh current densities (Figure S6). Furthermore, LOBs equipped with Fine Slab/Ti_4_O_7_@TiC catalyst demonstrate exceptional cycling stability exceeding 50 cycles (equivalent to 60 h) and a minimal overpotential (∼1.55 V) at 3000 mA g^−1^ in Figure 3h, despite ultra‐high current charging‐discharging procedures (10 000 mA g^−1^). The rate curve validates that C/Ti_4_O_7_ in‐situ synthesized on the surface could serve as an indispensable protective barrier that effectively mitigates structural degradation and maintains catalytic stability for whole hybrid. Owing to the unique crystal structure and electronic properties, the TiC core plays a pivotal role by providing abundant active sites for electrochemical reactions, thereby significantly enhancing charge transfer and reaction kinetics [30, 31, 32]. The Fine Slab/Ti_4_O_7_ shell optimizes overall performance through two primary mechanisms: strategically modifying the pore structure to facilitate ion transport and increasing the contact area between the electrolyte and the active material. The shell significantly enhances the electron transmission rate within Ti_4_O_7_. Moreover, the residual fine carbon is integral to effectively modify the electron cloud distribution in Ti_4_O_7_@TiC, thereby enhancing the migration and distribution of electrons and comprehensively.

### 2.3 Role of Ternary Heterointerface and Catalytic Mechanism

**FIGURE 4:**
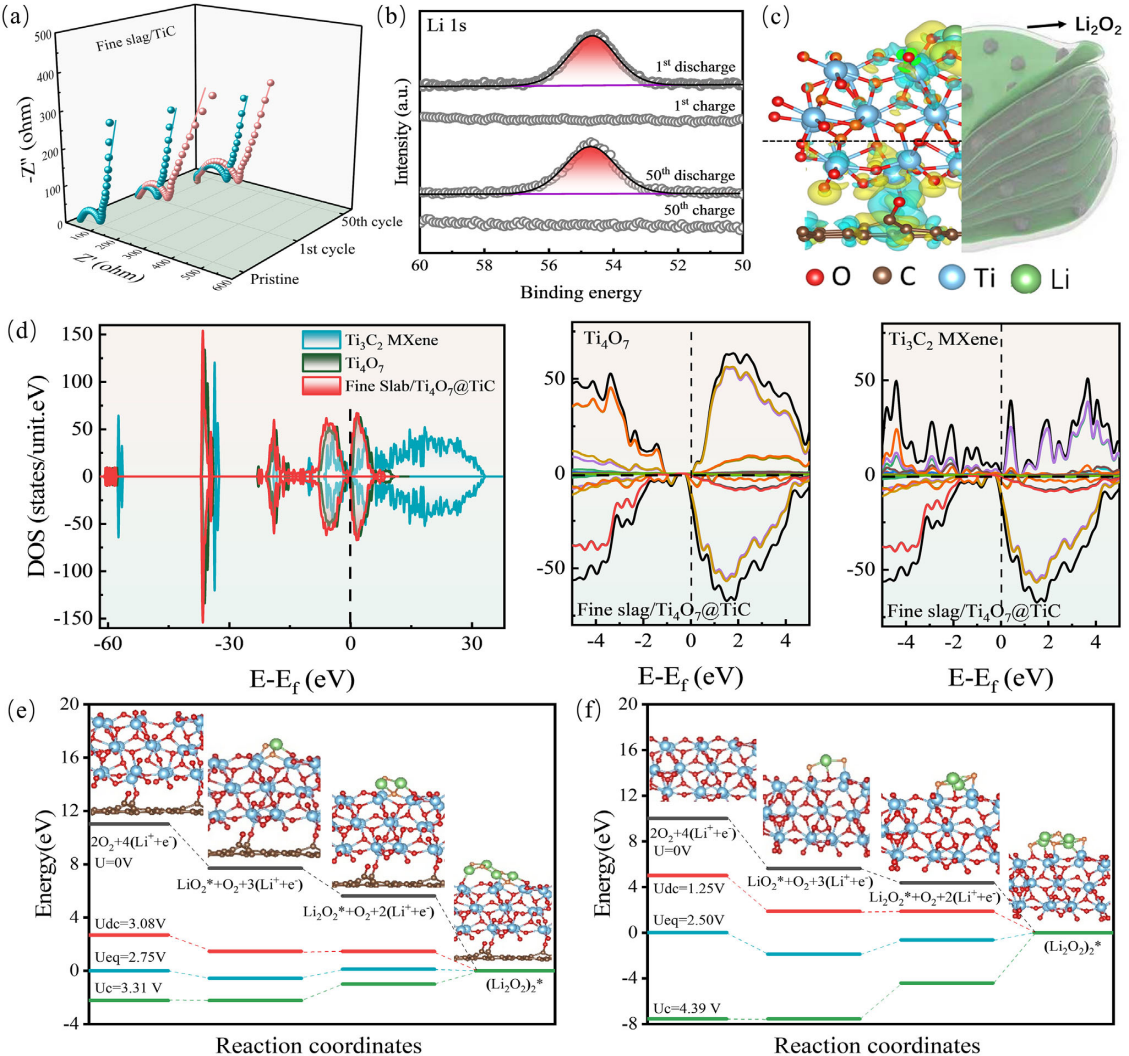
(a) EIS spectra of Fine Slab/@TiC‐based LOBs after different discharge–charge cycles (Pink curves represent the impedance data during the discharge process, while green curves represent the impedance data during the charge process); (b) Li 1s XPS spectra of Fine Slab/Ti_4_O_7_@TiC at selected discharge and charge states; (c) Charge density difference plots of Fine Slab/Ti_4_O_7_@TiC (yellow denoting the increased charge and cyan the decreased charge); (d) Electronic density of states (DOS) for the bulk structure of catalysts; Free energy diagrams of battery reactions on the surface of Fine Slab/Ti_4_O_7_@TiC (e) and Ti_4_O_7_ MXene (f).

Electrochemical impedance spectroscopy (EIS) was employed to quantify the charge transfer resistance and double‐layer capacitance at catalysts in LOBs (Figure 4a; Figure S7). The corresponding equivalent circuits are depicted in Figure S8, in which R_0_, CPE, *R*_ct_, and W_0_ denote the solution resistance, the double‐layer capacitance, charge‐transfer resistance, and diffusion resistance, respectively. The appearance of the CPE and *R*_ct_ parameters following discharge indicates the formation of a novel interface, which is associated with the generation of discharge by‐products. Within the equivalent circuit, the parallel arrangement of the CPE and *R*_ct_ could signify the existence of an interface. The pristine LOBs with Fine Slab/Ti_4_O_7_@TiC exhibit double parallel configuration of CPE and *R*_ct_, which provides indirect evidence for the presence of the carbon/Ti_4_O_7_ and TiC interfaces. The charge‐transfer resistance (*R*_ct_) of the Fine Slab/Ti_4_O_7_@TiC catalyst in LOBs delivers a significant increase from 79.81 (pristine) to 111.14 ohms (first discharge), suggesting a substantial accumulation of insulating products with low conductivity on the cathode surface. Remarkably, the *R*_ct_ was reduced to 79.23 ohms during the subsequent charging, suggesting that the low conductivity products formed during the discharge could be decomposed in charging process. Furthermore, the impedance of Fine Slab/Ti_4_O_7_@TiC after 50 charge‐discharge cycles exhibited a minimal rise (5.67 ohms) relative to the initial charge, indicating that the hybrid catalyst exhibits very exceptional stable stability during prolonged high‐rate cycling conditions. Ex‐situ XPS analysis in Figure 4b confirms that the formed product is lithium peroxide (Li_2_O_2_, 54.69 eV). This discharge product could be complete decomposed during the subsequent charging process. Meanwhile, the discharge product remains Li_2_O_2_ and retains its capability of decomposing fully during charging even after 50 cycles. Figure S9‐resolution TEM images clearly reveal the dynamic evolution of Li_2_O_2_ deposition morphology. After the first cycle, the discharge product exists in the form of discrete nanoparticles or thin layers, which is beneficial to maintain the efficient mass transfer and charge transfer at the electrode/electrolyte/Li_2_O_2_ three‐phase interface. By the 10th cycle, Li_2_O_2_ has evolved into a continuous and dense film‐like structure, most of which covers the surface of the Fine Slab/Ti_4_O_7_@TiC electrode, by the 50th cycle, Li_2_O_2_ has completely covered the surface of Fine Slab / Ti4O7 @ TiC electrode, significantly reducing the electrochemically active interface area. Figure Figure S9 High‐resolution TEM images reveal the dynamic evolution of the Li_2_O_2_ deposition morphology. After the first cycle, the discharge products exist as discrete nanoparticles or thin layers, which is beneficial for maintaining efficient mass and charge transfer at the electrode/electrolyte/Li_2_O_2_ triple‐phase interface. By the 10th cycle, Li_2_O_2_ evolves into a continuous and dense “film‐like” structure that covers most of the Fine Slab/Ti_4_O_7_@TiC electrode surface. Ultimately, by the 50th cycle, Li_2_O_2_ completely covers the electrode surface, significantly reducing the electrochemically active interface area [33, 34, 35, 36]. The characteristic membrane structure of Li_2_O_2_ is formed via a surface‐mediated growth mechanism, wherein the intermediate deposition of LiO_2_ plays a crucial role. Different from the traditionally observed toroidal morphology, the membranous form of Li_2_O_2_ offers a superior product/catalyst multiphase interface, which significantly enhances the kinetics of both ORR and OER.

O 1s XPS (Figure S9) provides a key chemical explanation for the morphology evolution. As the cycle progresses (1→10→50cycles), the signal attributed to Li_2_O_2_ (∼531.3 eV) is relatively weakened, while the signal representing Li_2_CO_3_ (∼531.8‐532.0 eV) is significantly enhanced. This indicates that the electrochemically reversible Li_2_O_2_ formed in the early stage is encapsulated by the side reaction product (Li_2_CO_3_) with stronger insulation and extremely slow decomposition kinetics in the subsequent cycle. Therefore, the “ film‐like product ” observed in the later stage is essentially an insulating composite layer with increasing Li_2_CO_3_ as the main body.

The intrinsic role of charge compensation arising from the interplay of oxygen vacancies and oxygen bridges in improving LOBs performance is elucidated by density functional theory (DFT). The DFT analysis reveals that C preferentially bonds with O atoms from Ti_4_O_7_ active layer of the Fine Slab/Ti_4_O_7_@TiC hybrid (Figure 4c), resulting in a diminished charge adsorption capacity oxygen (O). Specifically, when C atom is combined with O atoms from Ti_4_O_7_ layer on TiC, the charged areas between the C site and O atoms are observed to be smaller than those between the Ti site and O atoms (highlighted in cyan). The interface oxygen bridge between Ti and C facilitates the formation of chemical bonds and the transfer of electrons/ions between Ti_4_O_7_@TiC and amorphous carbon, thereby improving conductivity and chemical reactivity. The DFT results is consistent with previous XPS analysis, indicates that the Ti‐O‐C coupling is fabricated by the favorable bonding between foreign C atom and Ti_4_O_7_ active layer. Moreover, Figure 4d confirms that Ti_4_O_7_ exhibits an exceptional metallic conductor character, while Ti_3_C_2_ MXene behaves as a semiconductor with little bandgap. By comparison, Fine slab/Ti_4_O_7_@TiC hybrid could also manifest metallic conductivity the by the protective C/Ti_4_O_7_ shell. Uniformly dispersed carbon nanoparticles and in‐situ formed Ti_4_O_7_ can effectively modulate the electronic properties, thereby enhancing the conductivity and catalytic performance of the Fine Slab/Ti_4_O_7_@TiC hybrid. Figure 4e,f depict the detailed atomic‐level reaction pathways of Li_2_O_2_ product on the surfaces of Fine Slab/Ti_4_O_7_@TiC and pure Ti_4_O_7_, respectively. The overpotential for the oxygen reduction reaction (ORR) is indicated by the potential difference U_0_ – U_dc_, while the overpotential for the oxygen evolution reaction (OER) is represented by U_C_—U_0_. A notable decrease in the OER overpotential is observed for the Fine Slab/Ti_4_O_7_@TiC composite when compared to the pristine Ti_4_O_7_ and the monomer Ti_3_C_2_ MXene (Figure S10). The enhanced OER catalytic activity of the Fine Slab/Ti_4_O_7_@TiC catalyst can be attributed to the electron‐withdrawing effect of the O centers, which is regulated by dioxygen bridge coupling. This effect increases the electron density and the decomposition kinetics of *Li_2_O_2_. Oxygen vacancies within the catalyst structure could act as electron capture sites and provide transport pathways, while the interface Ti‐O‐C bonding facilitate the connection between various active sites or phases. As charge compensation occurs between these entities, the electron transport capability of the dioxygen bridge coupling is augmented. Consequently, the number and reactivity of active sites are increased, and the adsorption and conversion of reaction intermediates are optimized.

## 3 Conclusions

Summarily, the interfacial dioxygen bridge coupling within the Fine Slag/Ti_4_O_7_@TiC hybrid has been effectively fabricated to optimize oxygen redox dynamics in LOBs. The synergy of carbon (extracted from fine slag) and Ti_4_O_7_ (the active termination of TiC) that linked by Ti‐O‐C units could endow the hybrid with a distinctive morphology, exceptional high‐rate stability, a favorable electronic structure, and high electrocatalytic activity. The LOBs equipped with the Fine Slag/Ti_4_O_7_@TiC catalyst demonstrate a low voltage polarization of 1.42 V, excellent cycle stability of 210 cycles at 3000 mA g^−1^, and superior rate performance ranging from 1000 to 10 000 mA g^−1^. DFT calculations and experimental studies confirm the crucial function of the interfacial dioxygen bridge coupling in maximizing the benefits of electronic structural tuning and regulating the formation mechanism of Li_2_O_2_. This study deeply revealed the vast potential of dioxygen bridge coupling in the practical development of cathode catalysts derived from mining waste for LOBs.

## 4 Experimental Section

### 4.1 Synthesis of Modified Fine Slag

1.5 g Sodium carbonate (Na_2_CO_3_) powder was added into 2.5 g Coal Gasification Fine Slag stirring for 0.25 h. The mixture was transferred and sealed into graphite sagger and heated at 875°C for 2.5 h. The product was ground for 15 min and then 20 mL hydrofluoric acid solution (HF) was added into this product. The product was centrifuged with deionized water and ethanol at 8000 rpm for 4–5 times until the pH value was adjusted to ∼7 while removing excess impurities. Finally, the collected precipitation was dried at 60°C to obtain desirable Modified Coal Gasification Fine Slag.

### 4.2 Synthesis of Ti_3_C_2_ MXene

2 g Lithium fluoride (LiF) powder was added into 40 mL hydrogen chloride (9 M) solution under magnetic stirring for 0.5 h at room temperature to generate hydrofluoric acid solution (HF). Then, 2.0 g Ti_3_AlC_2_ MAX powder was added slowly into 50% HF for 1 h at 40°C under vigorous stirring. The mixture was transferred and sealed into 100 mL Teflon‐lined hydrothermal autoclave and heated at 90°C for 20 h. The product was centrifuged with deionized water and ethanol at 8000 rpm for 4–5 times until the pH value was adjusted to ∼7 while removing excess impurities. Finally, the collected precipitation was dried at 60°C and was calcined at 350°C for 2 h to obtain desirable Ti_3_C_2_ MXene.

### 4.3 Synthesis of Fine Slag/Ti_4_O_7_@TiC

0.15 g glucose and appropriate amount of modified coal gasification slag were dissolved in hydrofluoric acid solution (HF) with continuous magnetic stirring. Then, 0.2 g prefabricated Ti_3_AlC_2_ MAX was added into the above dispersion with continuous physical stirring. Then, the homogeneous solution was transferred and sealed into 100 mL Teflon‐lined hydrothermal autoclave and heated at 150°C for 18 h. The resultant mixture was washed by centrifuged with deionized water and ethanol to obtain precursor. Finally, the precursor was dried at 60°C to obtain the ideal Fine Slag/Ti_4_O_7_ @ TiC. The Coal Gasification Fine Slag nanoparticles were obtained by Hydrothermal reaction without the addition of Ti_3_AlC_2_ MAX under the same conditions as above.

The catalyst materials (Fine Slag/Ti_4_O_7_@TiC, Ti_3_C_2_ MXene, modified Fine Slag), conductive additive (acetylene black), and binder (polyvinylidene fluoride, PVDF) were weighed in a mass ratio of 7:2:1. After mixing and grinding, the mixture was dissolved in N‐Methyl pyrrolidone and stirred for 12 h to form a thick and uniform slurry. Then, the prepared slurry was coated onto the carbon paper and dried at 60°C. Finally, the catalyst‐based cathode with loading of ∼0.6 m  cm^−2^ was cut into a disk (ɸ = 12 mm). The 2032 coin‐type LOBs were assembled in a glovebox with as prepared cathode, Li metal anode, glass microfiber diaphragm (Grade GF/D), and electrolyte (1.0 M lithium bis (trifluoromethane sulfonyl) imide (Figure S1) dissolved in dimethyl sulfoxide). The assembled LOBs are placed in a self‐made glass bottle sealed with oxygen. The performance measurement of LOBs was conducted by the Blue Electric Battery Testing System (LANHE CT3001A) of Wuhan Blue Electric Company.

## Author Contributions

**J. Zhang**: conceptualization, data curation, writing – original draft preparation. **S. Ma**: methodology, visualization. **Z. Sun**: supervision, conceptualization, funding acquisition, writing – review & editing. **Y. Wang**: methodology. **H. Liu**: validation. **B. Huo**: methodology, validation. **K. Zeng**: writing – review & editing, funding acquisition, conceptualization.

## Conflicts of Interest

The authors declare no conflicts of interest.

## Supporting information




**Supporting File**: advs73556‐sup‐0001‐SuppMat.docx.

## Data Availability

Research data are not shared.
