# Supplementary material for: Enhancing Interfacial Dioxygen Bridging Dynamics of Waste‐Derived Cathode Catalysts for Augmented High‐Rate Performance in Li‐O2 Batteries
Source: Adv Sci (Weinh). 2026 Jan 12;13(14):e22315. doi: 10.1002/advs.202522315 (PMC12970270; doi:10.1002/advs.202522315)
Supplement: Supplementary file 1 — Supporting File: advs73556‐sup‐0001‐SuppMat.docx. [file ADVS-13-e22315-s001.docx]

Supporting Information

Enhancing Interfacial Dioxygen Bridging Dynamics in Waste-Derived Cathode Catalysts for Augmented High-Rate Performance in Li-O2 Batteries

Jixiong Zhang, Shuxuan Ma, Hengfeng Liu, Binbin Huo, Yunbo Wang*, Zhihui Sun*， and Kai Zeng*

Computational methods

DFT calculations were performed by using the Vienna Ab-initio Simulation Package (VASP).^[1]^ The exchange–correlation interactions were described by generalized gradient approximation (GGA)^[2]^ with the Perdew–Burke–Ernzerhof (PBE) functional^[3]^. Spin-polarization was included in all the calculations and a damped van der Waals correction was incorporated using Grimme’s scheme to better describe the non-bonding interactions.^[4]^ A plane wave cut-off energy of 500 eV was used, and a 2×2×1 Monkhorst-Pack grid k-points was employed. The residual force and energy on each atom during structure relaxation were converged to 0.005 eV A^-1^ and 10^-5^ eV, respectively. We simulated the Li_x_O_y_ intermediate adsorption performance on Ti_4_O_7_, Ti_3_C_2_O_2_ and Fine Slag/Ti_4_O_7_@TiC surface. The adsorption energy was defined as equation (1)

$E_{ads} =E \left( substrate + {Li}_{x}O_{y} \right)-E \left( substrate \right)-E ({Li}_{x}O_{y})$ (1)

Where E (substrate + Li_x_O_y_) represented the sum energy of Li_x_O_y_ intermediate adsorbed on the MoO_x_@Ti_3_C_2_ MXene surface, E (substrate) was the energy of Fine Slag/Ti_4_O_7_@TiC surface and E (Li_x_O_y_) represented the energy of Li_x_O_y_ intermediate. In addition, the free energy of adsorbed state was revised by equation (2) as followed

$\Delta G=\Delta E +\Delta E_{ZPE} -T\Delta S$ (2)

Where ΔE is the adsorption energy of Li_x_O_y_ intermediate obtained by equation1, ΔE_ZPE_ is the difference corresponding to the zero point energy and ΔS is the entropy change of the adsorption reaction. In addition, T was set as 298K to revise the circumstance to ambient temperature.


**Figure S1**. Self-made testing device for Li-O_2_ battery.


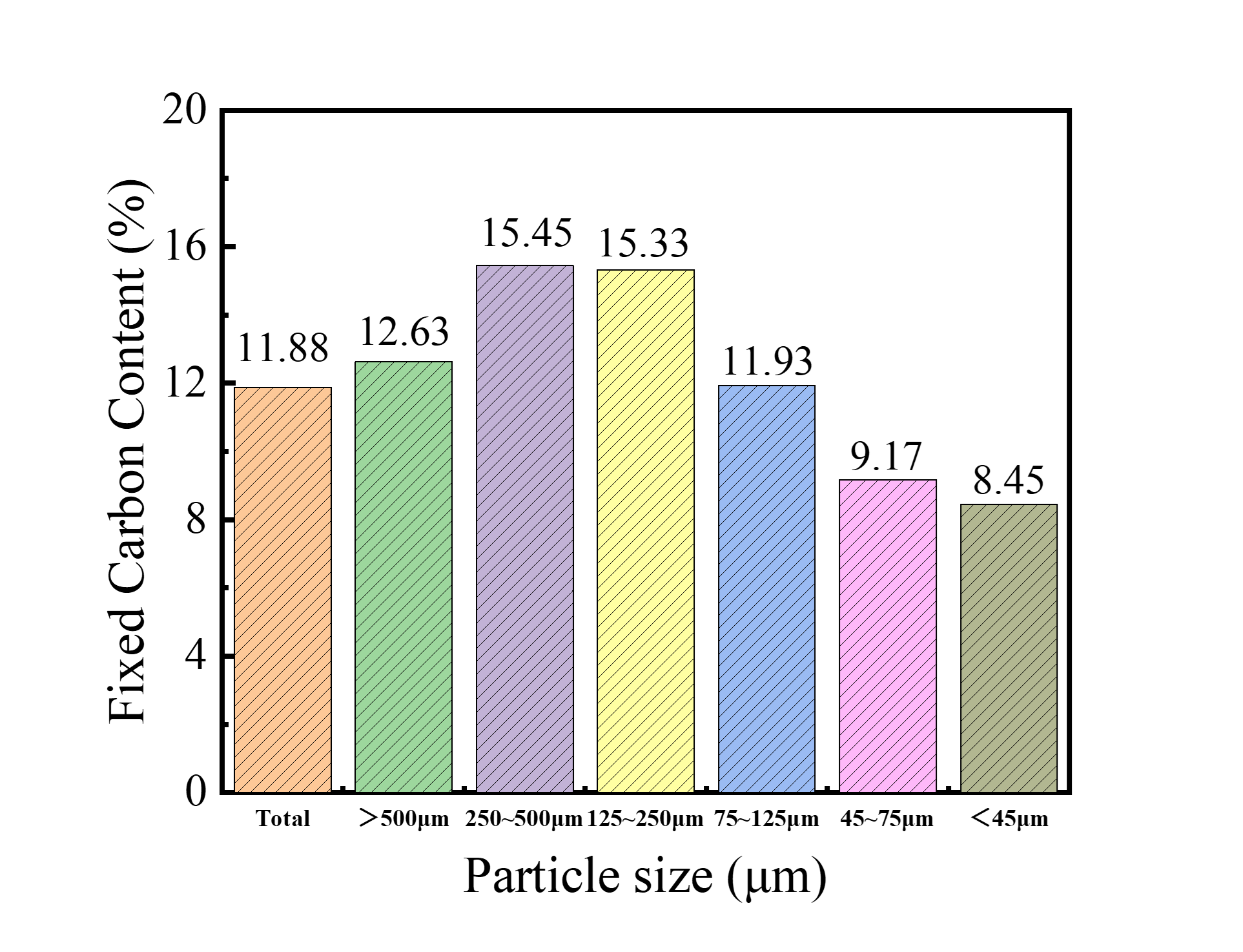


**Figure S2**. Analysis of fixed carbon content in coal gasification fine slag


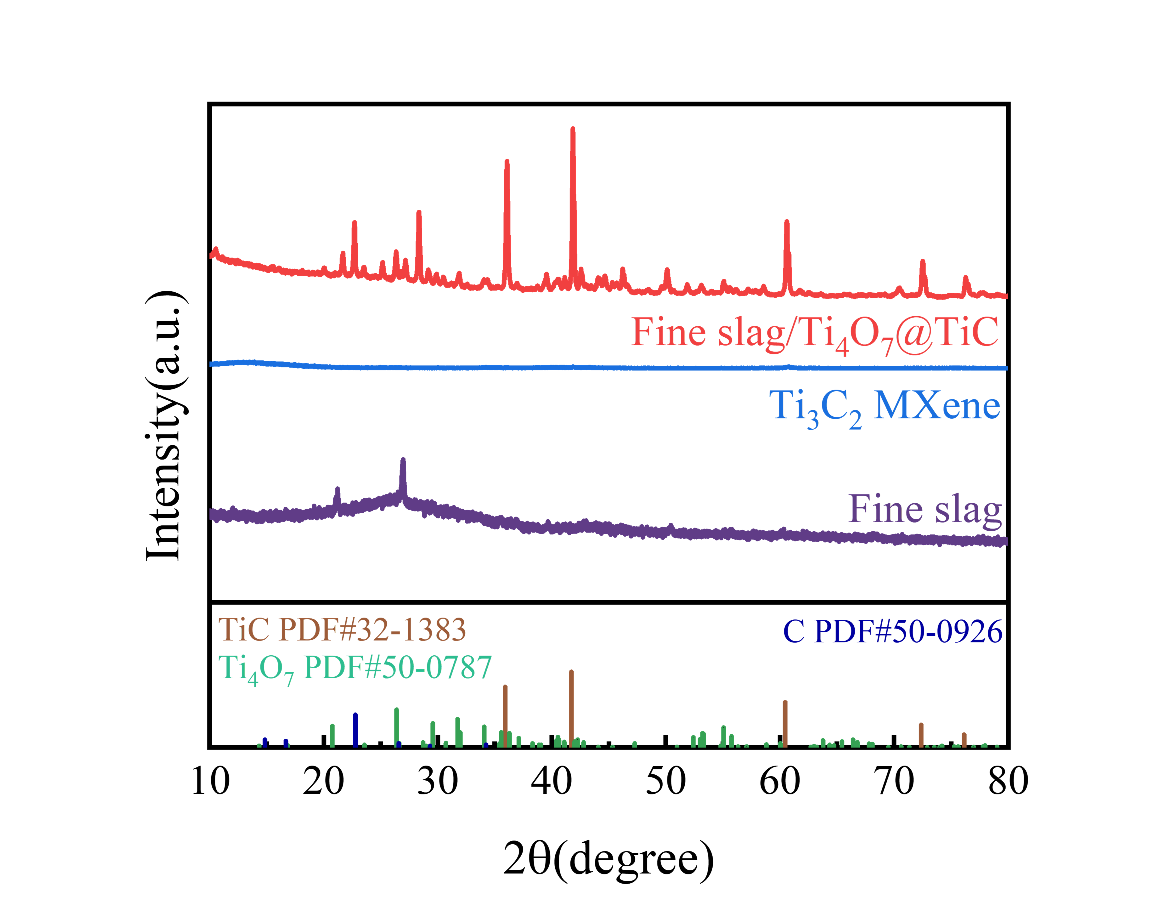


**Figure S3**. XRD patterns of the synthesized catalysts


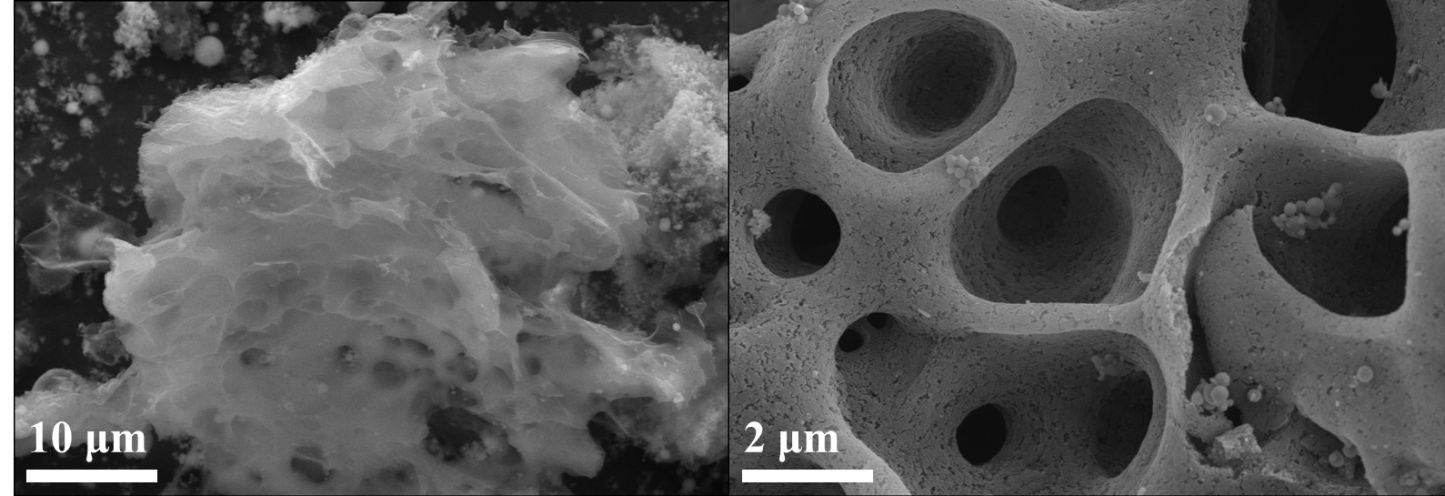


**Figure S4**. SEM image of pretreated fine slag.


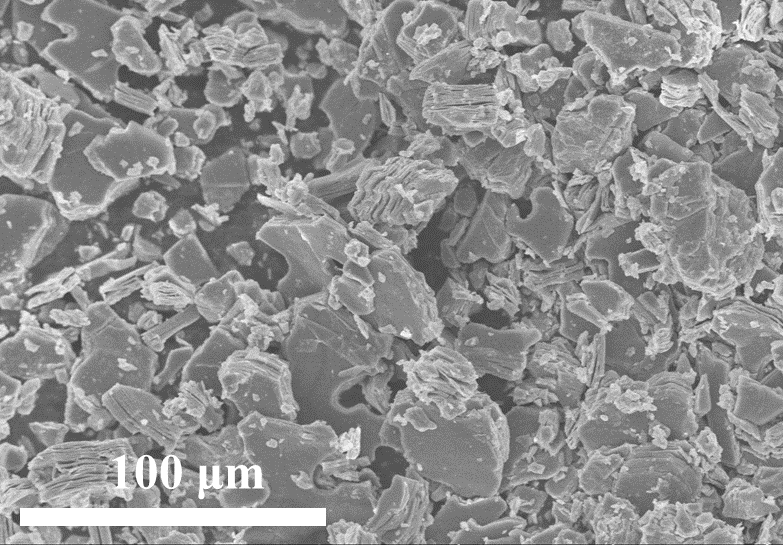


**Figure S5**. SEM image of Ti_3_C_2_ MXene.


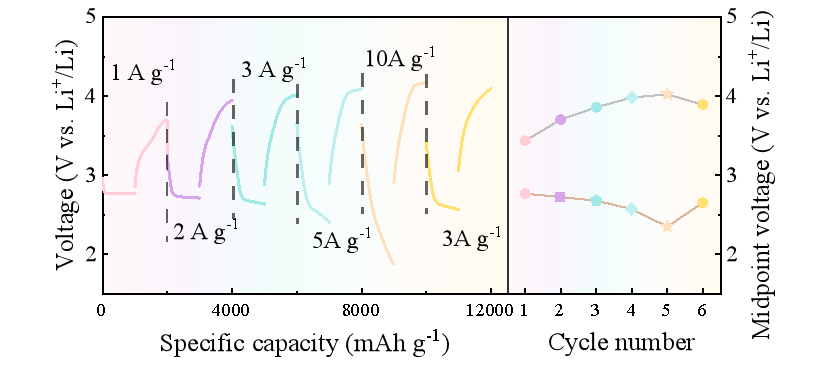


**Figure S6**. the rate capabilities of the MXene-based LOBs at varying current densities


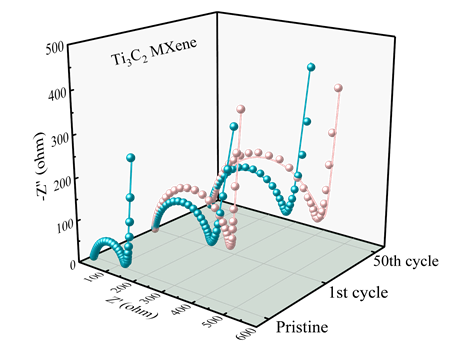


**Figure S7**. EIS spectra of Ti_3_C_2_ MXene.


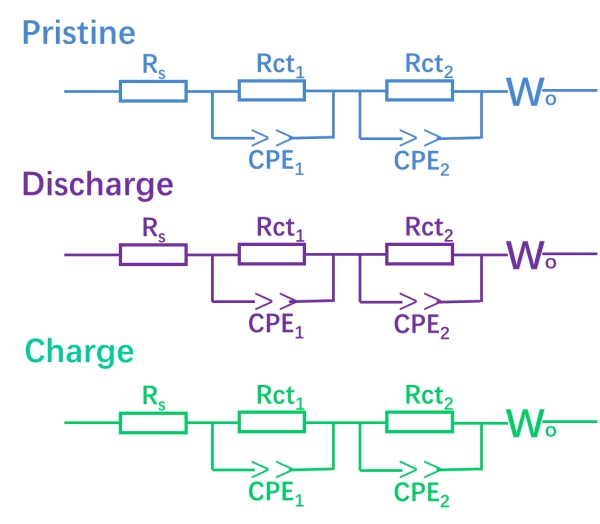


**Figure S8**. EIS corresponding analog circuit diagram.


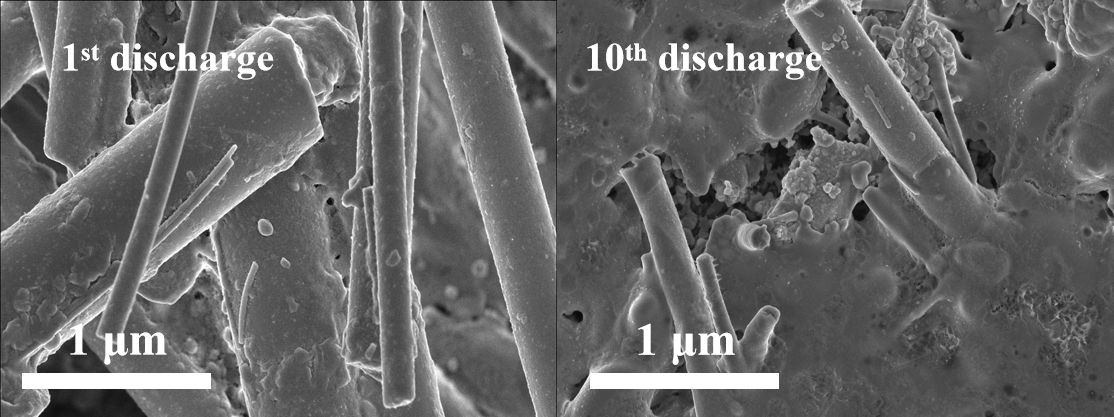


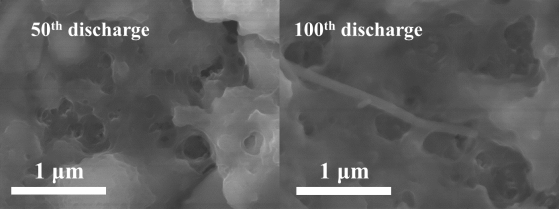





**Figure S9**. SEM images of Fine Slag/Ti_4_O_7_@TiC at different charging statuses and O 1s XPS images.


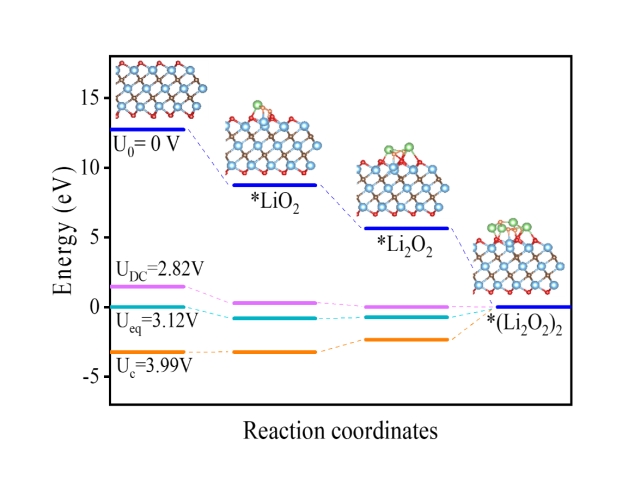


**Figure S10**. Free energy diagrams of battery reactions on the surface of MXene


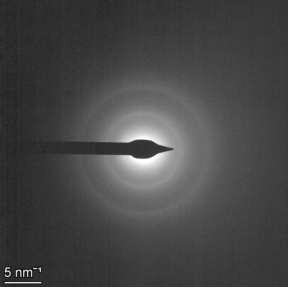


**Figure S11**. SAED of Fine Slag/Ti_4_O_7_@TiC





**Figure S12**. EDS spectra of Fine Slag/Ti_4_O_7_@TiC.





**Figure S13**.Discharge/charge at a current density of 3000 mA g^-1^ and a cut-off capacity of 1000 mAh g^-1^, first five cycles.

**Table S1**. Comparison of battery performance of **Fine Slab/Ti₄O₇@TiC** -based cathode with other reported electrodes.

| **material** | **Current density** | **Limitted specific capacity** | **Cycle number** | **Rf.** |
| --- | --- | --- | --- | --- |
| Ti_4_O_7_/MCO | 200 | 500 | 100 | 5 |
| Ru/Ti_4_O_7_ | 200 | 1000 | 100 | 6 |
| Ti_4_O_7_ | 100 | 500 | 70 | 7 |
| TiC/MWNTs-Ru | 250 | 1000 | 90 | 8 |
| TiC–C | 100 | 500 | 90 | 9 |
| Pt‒Ti_3_C_2_ | 500 | 100 | 110 | 10 |
| N-Ti_3_C_2_ | 500 | 200 | 372 | 11 |
| MoOx@Ti_3_C_2_ | 1000 | 3000 | 130 | 12 |
| NiO/Ti_3_C_2_ | 500 | 500 | 90 | 13 |
| Ti_0.87_O_2_/MXene | 600 | 1000 | 407 | 14 |
| Co@HCNs | 500 | 200 | 250 | 15 |
| Ni–Fe-NCNT | 1000 | 100 | 200 | 16 |
| RuCo-NC | 1000 | 500 | 298 | 17 |
| **Fine Slab/Ti₄O₇@TiC** | **3000** | **1000** | **210** | **This work** |

**Table S2**. Resistance value during the cycling process

| **State** | **R_1_** | **R_2_** | **R_3_** |
| --- | --- | --- | --- |
| Pristine | 11.08 | 5.99 | 73.82 |
| 1st discharge | 44.4 | 12.01 | 99.13 |
| 1st charge | 11.13 | 6.782 | 72.45 |
| 50th discharge | 43.43 | 20.37 | 100 |
| 50th charge | 11.09 | 9.846 | 63.71 |

**Table S3**. EDS elemental analysis of Fine Slag/Ti_4_O_7_@TiC gangue

| Element | Line Type | k Factor | Absorption Correction | Wt% | Atomic % |
| --- | --- | --- | --- | --- | --- |
| C | K series | 5.45 | 1.00 | 39.44 | 17.88 |
| F | K series | 4.18 | 1.00 | 17.37 | 12.46 |
| Na | K series | 2.47 | 1.00 | 2.85 | 0.53 |
| Mg | K series | 2.36 | 1.00 | 2.57 | 0.51 |
| Al | K series | 4.22 | 1.00 | 4.14 | 0.91 |
| Si | K series | 0.18 | 1.00 | 0.17 | 0.04 |
| Ti | K series | 6.30 | 1.00 | 33.46 | 60.44 |
| Total: |  |  |  | 100.00 | 92.77 |

**Reference**

1. Kresse G, Furthmüller J, “Efficiency of ab-initio total energy calculations for metals and semiconductors using a plane-wave basis set,” *Computational materials science* 6.1 (1996): 15-50.
2. Perdew J P, Burke K, Ernzerhof M, “Generalized Gradient Approximation Made Simple,” *Physical review letters* 77.18 (1996): 3865.
3. Perdew J P, Ernzerhof M, Burke K, “Rationale for mixing exact exchange with density functional approximations,” *The Journal of chemical physics* 105.22 (1996): 9982-9985.
4. Grimme S, “Semiempirical GGA-type density functional constructed with a long-range dispersion correction,” *Journal of computational chemistry* 27.15 (2006): 1787-1799.
5. Cao X, Sun Z, Zheng X, et al, “MnCo_2_O_4_ decorated Magnéli phase titanium oxide as a carbon-free cathode for Li-O_2_ batteries,” *Journal of Materials Chemistry A* 5.37 (2017): 19991-19996.
6. Cao X, Wei C, Zheng X, et al, “Ru clusters anchored on Magnéli phase Ti_4_O_7_ nanofibers enables flexible and highly efficient Li-O_2_ batteries,” *Energy Storage Materials* 50 (2022): 355-364.
7. Lee S, Lee G H, Kim J C, Kim D W, “Magnéli-Phase Ti_4_O_7_ Nanosphere Electrocatalyst Support for Carbon-Free Oxygen Electrodes in Lithium–Oxygen Batteries,” *ACS Catalysis* 8.3 (2018): 2601-2610.
8. Yang C S, Sun Z, Cui Z, Jianga FL, “Inward growth of superthin TiC skin on carbon nanotube framework as stable cathode support for Li-O_2_ batteries,” *Energy Storage Materials* 30 (2020): 59-66.
9. Qiu F, He P, Jiang J, Zhang X, “Ordered mesoporous TiC-C composites as cathode materials for Li-O_2_ batteries,” *Chemical Communications* 52.13 (2016): 2713-2716.
10. Cao D, Zheng L, Wang Y, et al, “Ultraviolet-assisted construction of low-Pt-loaded MXene catalysts for high-performance Li-O_2_ batteries,” *Energy Storage Materials* 51 (2022): 806-814.
11. Xu H, Zheng R, Du D, et al, “Adjusting the 3d Orbital Occupation of Ti in Ti_3_C_2_ MXene via Nitrogen Doping to Boost Oxygen Electrode Reactions in Li-O_2_ Battery,” *Small* 19.9 (2023): 2206611.
12. Sun Z, Hu Y, Zhang J, et al, “Interfacial oxygen bridge bonding with Mo-O-Ti units in MoOx@Ti_3_C_2_ MXene harness efficient Li-O_2_ Battery at high rate,” *Applied Catalysis B: Environment and Energy* 351 (2024): 123984.
13. Li X, Wen C, Yuan M, et al, “Nickel oxide nanoparticles decorated highly conductive Ti_3_C_2_ MXene as cathode catalyst for rechargeable Li-O_2_ battery,” *Journal of Alloys and Compounds* 824 (2020): 153803.
14. Zhang D, Zhang G, Liu R, et al, “Mutually Activated 2D Ti_0.87_O_2_/MXene Monolayers Through Electronic Compensation Effect as Highly Efficient Cathode Catalysts of Li-O_2_ Batteries,” *Advanced Functional Materials* 35.5 (2025): 2414679.
15. Yang X, Zhu J, Wang Y, et al, “Cobalt nanoparticles decorated hollow N-doped carbon nanospindles enable high-performance lithium-oxygen batteries,” *Journal of Colloid and Interface Science* 683 (2025): 926-933.
16. Lim Y, Chang H, Kim H, et al, “Sequential element control of non-precious dual atom catalysts on mesoporous carbon nanotubes for high performance lithium-oxygen batteries,” *Journal of Materials Chemistry A* 12.42 (2024): 28953-28964.

[17] Zhao Y, Meng K, Luo T, et al, “Electronic structure engineering of RuCo nanoalloys supported on nanoporous carbon for Li-O_2_ batteries,” *Journal of Power Sources* 597 (2024): 234130.
